# Assessment of heavy metals mobility and toxicity in contaminated sediments by sequential extraction and a battery of bioassays

**DOI:** 10.1007/s10646-015-1499-4

**Published:** 2015-06-10

**Authors:** Agnieszka Baran, Marek Tarnawski

**Affiliations:** Department of Agricultural and Environmental Chemistry, University of Agriculture in Krakow, Al. Mickiewicza 21, 31-120 Krakow, Poland; Department of Water Engineering and Geotechnics, University of Agriculture in Krakow, Al. Mickiewicza 24/28, 30-059 Krakow, Poland

**Keywords:** Heavy metals, Mobility, Toxicity, Sequential fractionation, Bioassays

## Abstract

The aim of this study was to assess heavy metals mobility and toxicity in sediments collected from a dam reservoir in the conditions of intensive human impact by using chemical fractionation and a battery of bioassays. In the studies, the test organisms were exposed to substances dissolved in water (Microtox, Phytotestkit) as well to substances absorbed on the surface of solid particles (Phytotoxkit, Ostracodtoxkit F). The studies showed that sediments from the Rybnik reservoir are toxic, but the tested organisms showed different sensitivity to heavy metals occurring in the bottom sediments. The sediment samples were classified as toxic and very toxic. Moreover, the studies showed a higher toxicity in solid phases and whole sediment than in pore water. The lowest sensitivity was observed in *H. incongruens* (solid phases*)* and *V. fischeri* (pore water, whole sediment). The studies revealed that the toxicity of the sediments is caused mainly by heavy metal forms associated with the solid phase of the sediments. The studies did not confirm the metals occurring in fraction I (exchangeable) to be bioavailable and toxic to living organisms because most correlations between the metal concentration in fraction I and the response of the organisms were negative. The highest mobility from the bottom sediments was found in zinc, average mobility—in copper, cadmium and nickel, and low mobility—in chromium and lead. Organic matter is likely to be the most important factor controlling metal distribution and mobility in the studied sediments.

## Introduction

Heavy metals constitute a significant part of sediment contaminants, which, at some concentrations, may be toxic to the aquatic ecosystem (Farkas et al. 2007; Du Laing et al. 2009; Christophoridis et al. 2009; Redriquez-Barroso et al. 2010; Tuikka et al. 2011). Total concentration of heavy metals might serve as useful indicators for appropriate assessment of sediment contamination. However, they cannot provide sufficient information to assess the environmental impact of contaminated sediments because metals are present in different chemical forms in sediments, which determines their mobility, potential toxicity and bioavailability (Morillo et al., 2004; Farkas et al. 2007; Shaheen and Rinklebe 2014; Rinklebe and Shaheen 2014). Several methods for determining different forms of metals in sediments are described (Tessier et al. 1979; Ure et al. 1993; BCR 2001). A sequential extraction method has been successfully applied to divide heavy metals in sediments into different binding forms: exchangeable, carbonate-bound, Fe–Mn oxide-bound, organic matter/sulfide-bound, and residual (Du Laing et al. 2009). Metals in exchangeable, carbonate-bound speciation are easily mobilizable fractions and are considered to be more mobile and bioavailable (MF—mobile fractions). The potential mobile fraction (PMF) of heavy metals including the non-residual fraction, i.e. carbonate, Fe–Mn oxides, and organic matter–bound fractions, can also be bioavailable if the properties of the sediment change (pH, redox potential) (Knox et al. 2006; Hunglei et al. 2008; Shaheen and Rinklebe 2014, Rinklebe and Shaheen 2014). Furthermore, chemical fractionation methods using several extractants do not give adequate information about metal bioavailability for all the metals present in a multi-contaminated sediment (Prokop et al. 2003). Many authors have shown that bioassays provide a general indication of metal bioavailability/toxicity in sediments (Latif and Licek 2004; Harikumar and Nasir 2010; Baran and Tarnawski 2013; Besser et al. 2014). Bioassays are a useful tool whose application enables a fuller classification of ecological risk resulting from the presence of chemical substances in sediments, their bioavailability, and interactions (Mankiewicz-Boczek et al. 2008; Nendza 2002; Davoren et al. 2005; Narracci et al. 2009; Buitrago et al. 2013). Many authors emphasize that bioassays are a good complement to chemical analyses in procedures of sediment quality assessment (Wadhia and Thompson 2007; Mamindy-Pajany et al. 2011). Due to the fact that organisms differ in sensitivity to various substances, it is essential to select appropriate test organisms. It is important for organisms to belong to different taxonomic groups and represent different links of the trophic chain.

The aims of these studies were: (1) to investigate the mobility of metals in sediments collected from a dam reservoir by using chemical fractionation; (2) to use four bioassays (Phytotoxkit, Phytotestkit, Ostracodtoxkit F, and Microtox^®^) to evaluate the toxicity of sediments, sediment elutriates and pore water, (3) and to analyze a possible relationship between the observed toxicity and mobility. The obtained information may provide a better understanding of environmental risks of heavy metals in sediments.

## Materials and methods

### Study area

**Fig. 1:**
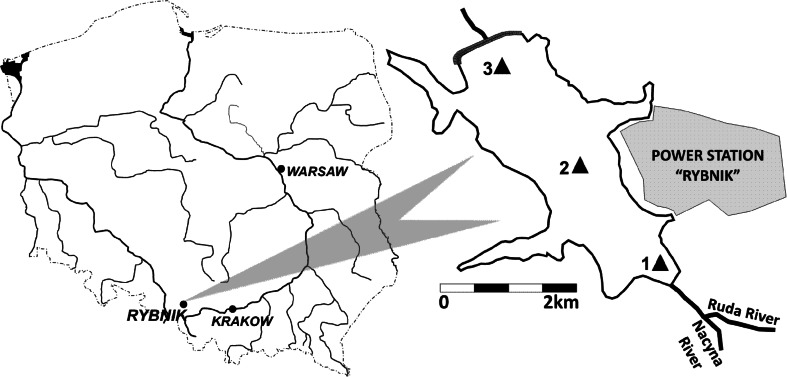
Bottom sediments’ sampling site

The Rybnik dam reservoir was built in 1972 for the needs of the Rybnik power plant S.A. (EDF) as a result of dividing the valley of the Ruda River (Odra River tributary) with an earth dam. The reservoir is one of the most important ones in the Silesian area and is the only anthropogenic-type reservoir in Poland (Fig. 1). This reservoir is part of a technological system as the source of water used in the power plant for cooling power facilities. The area of the main reservoir is 465 ha, and the total area along with lateral lakes is 555 ha. The capacity of the main part of the reservoir is 21.4 million m^3^, and the total capacity amounts to 24 million m^3^, at depths between 2 and 11 m. The length of the reservoir is approx. 4.5 km. The areas of the watershed that feed the reservoir as well as riparian areas have very varied management: industrial areas (plants and slag heaps), urban and rural development, forest complexes (coniferous and deciduous). The reservoir is located in one of the most industrialized areas in Poland (the *Upper Silesian District*), which affects the contamination of the reservoir. The Upper Silesian District is a region with an enormous concentration of industry, mainly hard coal mining and the electric power industry, hence strong attention is directed to the studies of Upper Silesian area surface water contamination (Koniarz et al. 2014).

### Sample collection

The samples were collected using an Ekman sampler from three set locations (Fig. 1). The top layer of the sediment was collected from 0-15 cm. Pore water for testing was isolated from the whole sediment by centrifugation (3000 rpm, 30 min.), filtered on a 0.45 µm filter, and placed in 50 cm^3^ polypropylene conical test tubes. The pore water samples were stored in the dark at 4 °C until they were used in the toxicity test. All the sediment samples after the decantation of overlying water and isolation of pore water were refrigerated until analyzed.

### Chemical analyses

The sediments were analyzed for parameters such as granulometric composition, pH, conductivity, organic matter, total heavy metal concentration. The pH was measured at a 1:2.5 sediment: liquid ratio with 1 mol KCl dm^−3^. Organic matter concentration was determined by loss-on-ignition for 8 h at 450 °C. Total element concentration in the sediments was assessed after hot digestion in a mixture of HNO_3_ and HClO_3_ (3:2 v/v) acids (suprapure, MERCK). Heavy metal concentrations were analyzed using ICP-AES method (Inductively coupled plasma atomic emission spectroscopy) on Optima 7300 DV (Perkin-Elmer). The speciation analysis of metals was performed using the three-step method of sequential fractionation by means of the modified BCR technique: fraction I—exchangeable and acid-soluble fraction, extractable with CH_3_COOH at 0.11 mol dm^−3^ concentration and pH 2; fraction II—forms associated with free Fe and Mn oxides, extractable with NH_2_OHHCl at 0.5 mol dm^−3^ concentration and pH 1.5; fraction III—forms bound to organic matter, extractable with hot 30 % H_2_O_2_, and then the mineralization products re-extracted with CH_3_COONH_4_ at 0.5 mol dm^−3^ concentration and pH 2. The residue (fraction IV) from step 3 was hot digested in a mixture of HNO_3_ and HClO_3_ acids (3:2) v/v. After each step the extracts were separated from the solid residue by centrifugation at 3000 rpm for 20 min, and the supernatant liquids were decanted into a polyethylene container. The extracts were stored in a refrigerator at about 4 °C prior to the analysis. The residues were washed by adding 20 ml of distilled water, shaken for 15 min on an end-over-end shaker, and centrifuged for 20 min at 3000 rpm. Metal concentrations in the obtained solutions were assessed using ICP-AES method on Optima 7300 DV PerkinElmer.

Accuracy of the performed analyses was tested using reference material CRM 16-050. The sediment and all pore water samples were analyzed in three replicates for which the relative standard deviations (%RSDs) were less than 10 % for all metals. The analytical results of the quality control samples showed good agreement with the certified values, recoveries ranging 93.6 (Cd)–105.4 % (Ni). The recovery of metal reached by sequential extraction technique was assessed by comparing the sum of the metal extracted in four steps (∑ fractions I–IV) with total metal concentration, using a mixture of HNO_3_ and HClO_3_ (3:2 v/v) acids for each sample. The results showed that the percentage of recovery ranged from 85 to 129 % for Zn, from 79 to 105 % for Cu, from 94 to 123 % for Pb, from 75 to 97 % for Cr, from 95 to 110 % for Cd, and from 90 to 105 % for Ni. The results were verified statistically using the Statistica 10 software package.

#### Evaluation of the environmental significance of metals in sediments

To assess metal concentrations in sediment, two guidelines were applied in these studies. The assessment of bottom sediment contamination with heavy metals was based on threshold effect concentration (TEC) and probable effect concentration (PEC) methods (Macdonald et al. 2000). These indices establish values which are to be considered as a threshold value of TEC as well as a probable value of PEC (Table 2). The sediment samples were predicted to be non-toxic if the measured concentrations of a chemical substance were lower than the corresponding TEC. Similarly, the samples were predicted to be toxic if the corresponding PECs were exceeded in the field-collected sediments. Samples with contaminant concentrations between the TEC and PEC were predicted to be neither toxic nor non-toxic (Macdonald et al. 2000). The other guideline was risk assessment code (RAC) classification based on the percentage of metal in the exchangeable and acid-soluble fraction (fraction I) (Singh et al. 2005). Risk Assessment Code indicates: no risk < 1 %; low risk 1-10 %; medium risk 11-30 %; high risk 31-50 %; very high risk > 50 %. The RAC classification was used to assess the risk connected with the release of heavy metals from the sediments.

### Ecotoxicity tests

In the first stage, a battery of screening bioassays was conducted on the sediment and pore water. The toxicity assessment was performed using the following tests: Phytotoxkit (sediment), Phytotestkit (pore water), Ostracodtoxkit F (sediment), and Microtox^®^ (sediment elutriate, pore water) (Table 1). Phytotoxkit and Phytotestkit, applied for the sediment and pore water toxicity classification, use three plants: *Sorghum saccharatum*, *Lepidium sativum,* and *Sinapis alba*. The measured parameters were inhibition of seed germination (IG) and root length inhibition (IR) in the tested sediment/pore water in comparison with the control sediment/redistilled water. The direct contact Ostracodtoxkit F microbiotest was used to measure the mortality and growth inhibition of neonates of the benthic ostracod crustacean *Heterocypris incongruens* hatched from cysts after 6 days of exposure to the sediment samples. After 6 days of contact with the sediment, the percentage mortality and growth of the crustaceans were determined and compared with the results obtained in the (non-toxic) reference sediment. The sensitivity of this 6-day biotest was compared with the 10-day amphipod crustacean test *Hyalella azteca* and 10-day assay with the midge larva *Chironomus riparius* (Chial and Persoon 2003). The results show that Ostracodtoxkit, in addition to having the advantages of stock independence, user-friendliness, and cost-effectiveness, appeared to perform as well as two other direct contact assays in detecting and quantifying the sediment toxicity (Chial and Persoon 2003). Ostracodtoxkit F is the very first “sediment contact” microbiotest with a crustacean test species for the assessment of the “total” toxicity of sediments, hence including the toxic hazard of both dissolved and non-dissolved contaminants (Ostracodtoxkit F 2001, ISO 14371:^2012^). In the Microtox^®^ test, the toxicity level of the samples was determined by a decrease in luminescence in *Vibrio fischeri*, which is an effect of metabolic inhibition in the bacteria after exposure to a toxic substance. The same standard test procedure was applied for sediment elutriate and pore water samples: 81.9 % Screening Test. The analysis of measurement of the change in luminescence was performed on a Microtox M500 Analyzer (MicrobicsCorporation 1992). The sediment elutriate was prepared by mixing one volume of the sediment with four volumes of redistilled water and shaking mechanically for 24 h (Baran and Tarnawski 2013). After that time, the samples were centrifuged for 10 min at a speed of 3000 rpm and filtered. Luminescence was measured before and after 15 min of incubation of the bacterial suspension with the studied sample. The tests were conducted in accordance with the procedure recommended by the manufacturer (MicrobicsCorporation 1992; Ostracodtoxkit 2001; Phytotoxkit 2004). Three replicate samples were tested. Toxicity results were expressed as percent effect (PE %).

**Table 1:** Battery of bioassays

Trophic level	Organisms	Test	Test reaction	Time
Producers	*S. saccharatum*, *L. sativum*, *S. alba*	Phytotoxkit/Phytototeskit	Germination and growth inhibition	72 h
Consumers	*H. incongruens*	Ostracodtoxkit F	Mortality, growth inhibition	6 days
Decomposer	*V. fischeri*	Microtox^®^	Luminescence inhibiotion	15 min.

In the second stage, after determining the percent effect for each bioassay, the sample was classified into one of five classes according to the highest toxicity indicated by at least one test: class I—no acute toxicity PE < 20 %; class II—slight acute toxicity 20 % ≤ PE < 50 %; class III—acute toxicity 50 % ≤ PE < 75 %; class IV—high acute toxicity 75 % ≤ PE < 100 %; class V—very high acute toxicity PE ≥ 100 % (Persoone et al. 2003; Matejczyk et al. 2011; Foucault et al. 2013).

## Results

### Physico-chemical properties of bottom sediments

The basic physico-chemical properties of the bottom sediments are presented in Table 2. The sediments showed a slightly acid reaction, and pH was within the range from 6.0 to 6.7. Electrolytic conductivity was between 1.1 and 1.6 mS cm^−1^. The concentration of organic matter in the bottom sediments was high and within the range from 257.8 to 287.6 g kg^−1^. Based on grain composition, bottom material from the Rybnik reservoir was classified as sandy loam (sample 1); sandy clay (sample 2); light loam (sample 3).

**Table 2:** Maine physicochemical characteristics of the sediments

Samples	Granulometric (%)			pH	Org. matter (g kg^−1^)	EC (mS cm^−1^)
	Sand	Silt	Clay	KCl		
1	34	43	23	6.0	257.8	1.1
2	54	22	24	6.2	287.6	1.6
3	37	38	25	6.7	250.0	1.1

Total heavy metal concentration in bottom sediments (mg kg^−1^ d.m.)						
Samples	Zn	Cu	Pb	Cd	Ni	Cr
1	1033	140	77.6	6.0	32.4	137
2	902	681	73.7	16.8	47.3	125
3	1077	110	125	5.0	28.6	197
TEC/PEC	121/459	31.6/149	35.8/128	0.99/4.98	22.7/48.6	43.3/111

The total heavy metal concentration in the sediments was between 902 and 1077 mg Zn; between 110 and 681 mg Cu; between 110 and 688 mg Cd; between 73.7 and 125 mg Pb; between 5.0 and 16.8 mg Cd; between 28.6 and 47.3 mg Ni; and between 125 and 197 mg Cr kg^−1^ d.m. The highest Zn, Pb, Cr concentrations in the bottom sediments were found in point 3. The highest Cu, Cd and Ni concentrations were found in point 2. The metal concentrations at each point were compared with the sediment quality guideline values referred to as the TEC and PEC (Table 2). In the case of zinc, cadmium, chromium (all measuring points), and copper (point 2), PEC values were exceeded. TEC values were exceeded in the case of nickel, lead (all points) as well as copper (points 1 and 3).

**Fig. 2:**
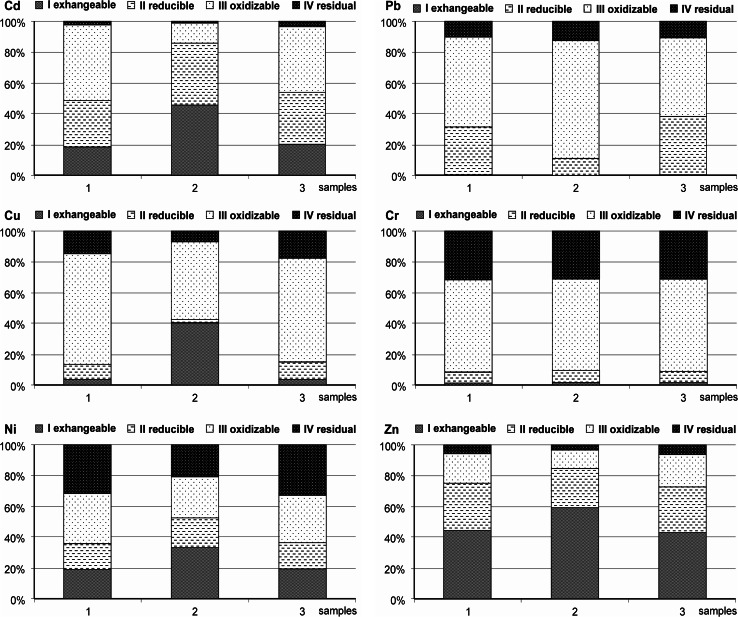
Fractional distribution and speciation of heavy metals in the sediment samples

Since mobility and potential toxicity of heavy metals are generally dependent on their existing chemical forms, it is important to identify the fractions of heavy metals in sediments. An analysis of data presented in Fig. 2 made it possible to evaluate the possibility for metals to migrate, and thereby to evaluate their potential toxicity to living organisms. The dominant zinc concentration was connected with fraction I (ion-exchangeable and carbonate, MF). Depending on the location of sediment collection, it was between 43 and 59 % of the total concentration of this metal in the sediments (Fig. 2). Fraction II (oxide) also played a considerable role in the binding of zinc (23–30 %). Zinc associated with fraction III (organic) and IV (residual) constituted, respectively, between 12 and 22 % and between 3 and 7 % of the total concentration in the sediments. Copper was present, above all, in fraction III (organic). Depending on the location of collection of the sediment samples, the percentage of copper in that fraction was between 51 and 85 % of its total concentration (Fig. 2). This may be due to the high affinity of Cu to organic matter (Shaheen and Rinklebe 2014). Copper associated with fraction I (mobile fraction MF) constituted between 1 and 41 %, with fraction II—between 2 and 12 %, and with fraction IV—between 7 and 18 %. Cadmium was associated with different fractions depending on the location of sediment collection. In the case of the sediment from measuring points 1 and 3, fraction III (49 %, 43 %) was dominant, and in the case of point 2—fraction I (46 %). Fractions I (18–46 %) and II (21–40 %) also played a considerable role in the binding of cadmium. Cadmium associated with fraction IV constituted only 2–3 % of the total concentration of this metal. These results show that the potential mobility of cadmium in the sediment is high (PMF ∑ 95–98 %), and thus, may result in a transfer into water. Similarly to copper, lead was present, above all, in fraction III (51–78 %). Fraction III is very important for Pb distribution due to the high affinity of Pb to organic matter (Rinklebe and Shaheen 2014). Moreover, this metal was associated in significant part with iron oxides and manganese oxides (fraction II) between 4 and 37 % of its total concentration in the sediments. Scanty amounts of lead—up to 1 %—were connected with fraction I. Nickel concentration in individual fractions was very varied. Nickel associated with fraction I constituted between 19 and 34 %; with fraction II—between 14 and 19 %; with fraction III—between 27 and 33 %; and with fraction IV—between 21 and 33 % of its total concentration in the sediments. The dominant concentration of chromium, similarly to copper and lead, was associated with fraction III (60–61 %). What is more, chromium in significant amount was associated with fraction IV (30–31 %). Chromium associated with fractions II and I constituted, respectively, between 7 and 8 % and up to 1 % of the total concentration of this metal in the studied sediments. To summarize, the heavy metals associated with different fractions in bottom sediments follow the order: Zn: exchangeable (I) > reducible (II) > oxidizable (III) > residual (IV); Cu: oxidizable (III) > residual (IV) > exchangeable (I) > reducible (II); Cd: oxidizable (III) ≈ reducible (II) > exchangeable (I) > residual (IV); Pb: oxidizable (III) > reducible (II) > residual (IV) > exchangeable (I); Ni: oxidizable (III) ≈ residual (IV) > exchangeable (I) > reducible (II); Cr: oxidizable (III) > residual (IV) > reducible (III) > exchangeable (I). The PMF (∑1–3) ranged from 90 to 97 % Zn; from 82 to 93 % Cu; from 95 to 98 % Cd; from 88 to 90 % Pb; from 67 to 79 % Ni, and from 65 to 69 % Cr of the total concentration of the metals. However, the mobile fraction MF (fraction I) ranged from 43 to 59 % Zn; from 3 to 41 % Cu, from 18 to 46 % Cd; from 0.37 to 0.68 % Pb; from 19 to 34 % Ni; and from 0.89 to 1 % Cr of total concentration of the metals. The order of the PMF in the studied sediments was Cd > Zn > Pb > Cu > Ni > Cr, while the order of the MF (fraction I) was Zn > Cd > Ni > Cu > Pb > Cr.

It is evident from the results of the fractionation study (Fig. 2) that the metals in the sediments are bound to different fractions with different strength. The strength values can, therefore, provide a clear indication of sediment reactivity, which in turn assesses the risk connected with the presence of metals in the aquatic environment. Risk Assessment Code indicates that sediment which in fraction I can release less than 1 % of the total metal will be considered safe for the environment. A sediment releasing (in the same fraction) more than 50 % of the total metal has to be considered highly dangerous and can easily enter the water-food chain, and will be considered hazardous to the environment. According to the Risk Assessment Code, high and very high risk of zinc release from bottom sediments was found; high and medium risk of copper, cadmium, and nickel release; low risk of chromium release; and no risk in the case of lead. The highest mobility of zinc, cadmium, copper and nickel from the bottom sediments to the aquatic environment was found in point 2 (Fig. 2). Fractions are very useful not only for determining the degree of binding of heavy metals in sediments and to what extent they may be remobilized into the environment (Turki 2007), but also for distinguishing those metals of lithogenic origin from those of anthropogenic origin. Heavy metals of anthropogenic origin are considered to be present mainly in the first fraction, while in the residual fraction metals of lithogenic origin are present. The partitioning shows that the percentages of metals bound with the non-residual fractions were greater than those of the residual fraction (Fig. 2), suggesting that these metals are primarily derived from anthropogenic inputs rather than geochemical background.

### Sediment and pore water toxicity

The results of toxicity of the bottom sediments and pore water are presented in Tables 3 and 4. In Phytotoxkit test, germination inhibition of the test plants was between 0 and 89 % (solid phases), between 0 and 44 % (pore water), and between 0 and 20 % (whole sediment). Depending on the studied phase, root growth inhibition varied between 24 and 100 % (solid phases), between −2 and 86 % (whole sediment), and between −16 and 38 % (pore water). In Ostracodtoxkit F test, the *H. incongruens* mortality was between 0 and 45 %, whereas growth inhibition was within a range from 1 to 52 %. Luminescence inhibition of *V. fischeri* was between 62 and 93 % (solid phases), between 6 and 47 % (whole sediment), and between −63 and −33 % (pore water). Generally, Phytotoxkit and Microtox tests showed the highest toxicity in the solid phase of the sediments, and the lowest in pore water. On the other hand, Ostracodtoxkit F test showed that the mortality rate and growth inhibition of *H. incongruens* was higher in the whole sediment only after the decantation of overlying water than in the solid phase of the sediments (Table 3).

**Table 3:** Sediments and pore water toxicity for organisms (percent effect PE %)

Samples	Phytotoxkit							Ostracodtoxkit F		Microtox
	Germination inhibition			Roots growth inhibition				Mortality	Growth inhibition	Luminescence
	^1^Ls	Sa	Ss	Ls	Sa	Ss		*H. incongruens*		*V. fischeri*
Whole bottom sediments										
1	10	0	10	86	42	46		20	24	47
2	10	10	10	47	16	2		10	27	22
3	10	10	20	26	59	11		45	52	6
Solid phases of sediments										
1	79	70	30	24	33	24	0		12	93
2	79	40	0	89	72	45	0		1	63
3	89	80	30	100	100	70	0		13	62
Pore water										
1	31	10	44	18	12	13	–		–	−36
2	19	0	44	8	−5	−16	–		–	−63
3	31	0	0	8	−16	38	–		–	−33

*Ls*, *L. sativum*; *Sa*, *S. alba*; *Ss*, *S. saccharatum*

**Table 4:** Hazard classification

Samples	^a^Class	Maximum class weight	Class weight score in %	Toxicity
Whole bottom sediments (wet bottom sediment)				
1	IV	3	39	High acute toxicity
2	II	1	44	Slight acute toxicity
3	III	2	33	Acute toxicity
Solid phases of sediments (dry bottom sediment)				
1	IV	3	67	High acute toxicity
2	IV	3	67	High acute toxicity
3	V	4	70	Very high acute toxicity
Pore water				
1	II	1	29	Slight acute toxicity
2	I	1	14	No acute toxicity
3	II	1	29	Slight acute toxicity

^a^ Class: I no acute toxicity PE < 20 %; II slight acute toxicity 20 % ≤ PE < 50 %; III acute toxicity 50 % ≤ PE < 75 %; IV high acute toxicity 75 % ≤ PE < 100 %; very high acute toxicity PE ≥ 100 %

Solid phases of the sediments were classified into toxicity class V (point 3) and IV (points 1 and 2) (Table 4). The whole sediment samples showed class IV (point 1), III (point 3), and II (point 2). The pore water samples were the least toxic and classified into toxicity class I (point 2) and II (points 1 and 2). Toxicity of the studied samples can be put in the following order: solid phases > whole sediment > pore water. Sediment collected in point 2 showed the lowest toxicity to the test organisms (Table 4), and sediments collected in point 3 showed the highest toxicity (Table 4).

**Fig. 3:**
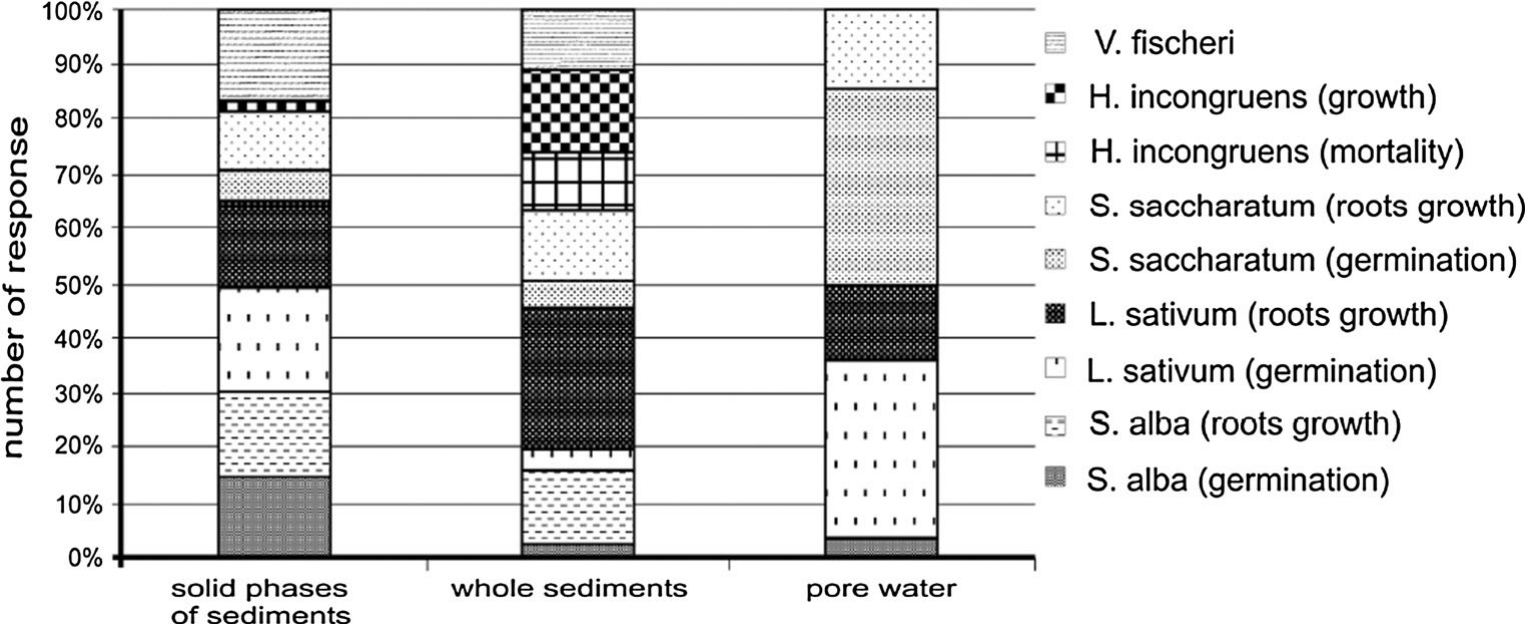
The number of response described for each applied microbiotest as the percentage from the total tests

Figure 3 presents sensitivity of the test organisms. While estimating the sensitivity of the performed bioassays, the highest number of toxic responses in the sediments was recorded for *L. sativum* (whole sediment, solid phases) as well as for *V. fischeri* and *S. alba* (solid phases), and in pore water—for *S. saccharatum* and *L. sativum* (Fig. 3). Among the plant species, *L. sativum* appears as the most sensitive (Fig. 3). This result is not in agreement with works by Czerniawska-Kusza et al. (2006) and Baran and Tarnawski (2013) who reported that *S. saccharatum* is the most sensitive species to identify phytotoxic sediment samples compared to *L. sativum* and *S. alba*. Among all the test organisms, the lowest sensitivity was observed in *H. incongruens* (solid phases) and *V. fischeri* (pore water, whole sediment). In studies undertaken by Mankiewicz-Boczek et al. (2008), the highest number of toxic responses was observed in the chronic Phytotoxkit test with higher plants, then in the Microtox^®^ test with *V. fischeri* bacteria, and in the Ostracodtoxkit F test with *H. incongruen*s.

### Correlation coefficient analysis

The correlation analysis performed on the data enabled the identification of possible common characteristics of heavy metals in the sediment, as well as evaluation of the potential of organic matter, pH and granulometric composition in order to control metal mobility. Results in Table 5 show that fraction I of Zn, Cu, Cd, Ni and Cr was positively correlated with sand and organic matter, while fraction I of Pb was positively correlated with clay and pH. Additionally, fraction I of Zn, Cu, Cd, Ni and Cr was negatively correlated with silt. Based on the correlation matrices obtained for faction II of the heavy metals, two clearly distinct metal groups may be distinguished: one for Zn, Pb, Ni, and another for Cu, Cd, Cr (Table 5). The first group of metals bound with faction II (forms associated with free Fe and Mn oxides) negatively correlated with sand and organic matter. However, they were positively correlated strongly with silt. The second group of metals showed the opposite correlations (Table 5). Data in Table 5 indicate that fraction III and fraction IV of the metals (Zn, Cu, Cd, Ni and Cr) showed a negatively significant correlation with sand and organic matter. However, these fractions correlated positively with silt. The PMF (∑I–III) of the metals (without lead) correlated positively with sand and organic matter, and negatively with silt (Table 5). Total Zn concentration was significantly correlated with sand (negatively), silt and organic matter (positively). Total concentration of Cu and Ni correlated positively with sand and organic matter. A strong negative relation was recorded between total concentration of both metals and silt (Table 5). A positive significant relation was found between total concentration of Cd and sand as well as clay. Total concentration of Pb and Cr showed a similar relation with properties of the sediments. A positive correlation was recorded between total concentration of both metals and clay, and pH (Table 5). However, total concentration of Pb and Cr was negatively correlated with organic matter. To sum up, the correlation analysis found that organic matter, sand and silt significantly affected the mobility and potential mobility of the metals (without lead). However, clay content and pH did not significantly affect the distribution of metals among different geochemical fractions. The lack of significantly correlation between the metal fractions and clay may be caused by the compositional variety of the clay fraction in the sediments, but might imply that clay minerals are only one of the factors controlling the fixation of heavy metals (Shaheen and Rinklebe 2014). Additionally, the lack of correlation between total concentration of Zn, Cu, Ni and clay may indicate that anthropogenic activities contribute as a source for these metals in sediment (Rinklebe and Shaheen 2014).

**Table 5:** Relationships between toxicity of sediments, sediments properties and metal fractions (% of total)

Parameters		IGe			IR			M	IGr	IL	Properties of sediement				
		*Sa*	*Ls*	*Ss*	*Sa*	*Ls*	*Ss*	*Hi*	*Hi*	*Vf*	Sand	Silt	Clay	pH	OM
MF fraction I	Zn	–0.98^***^	ns	–0.98^***^	ns	ns	ns	ns	–0.97^***^	ns	0.97^***^	–0.95^***^	ns	ns	0.98^***^
	Cu	–0.97^**^	ns	–0.98^***^	ns	ns	ns	ns	–0.97^***^	ns	0.98^***^	–0.99^***^	ns	ns	0.97^***^
	Pb	ns	ns	ns	0.81^**^	0.61^*^	0.89^**^	0.91^***^	ns	–0.62^*^	ns	ns	0.97^***^	0.88^**^	ns
	Cd	–0.91^**^	ns	–0.88^**^	ns	ns	ns	ns	–0.97^***^	ns	0.99^***^	–0.98^***^	ns	ns	0.98^***^
	Ni	–0.98^***^	ns	–0.97^**^	ns	ns	ns	ns	–0.97^***^	ns	0.99^***^	–0.97^***^	ns	ns	0.98^***^
	Cr	–0.74^**^	ns	ns	ns	ns	ns	ns	–0.83^**^	–0.84^**^	0.94^***^	–0.96^***^	ns	ns	ns
Fraction II	Zn	0.95^***^	ns	0.98^***^	ns	ns	ns	ns	0.99^***^	ns	–0.98^***^	0.99^***^	ns	ns	–0.97^***^
	Cu	–0.84^**^	ns	–0.94^***^	ns	ns	ns	ns	–0.92^***^	–0.74^**^	0.98^***^	–0.99^***^	ns	ns	0.86^**^
	Pb	0.98^***^	ss	0.97^***^	ns	ns	ns	ns	0.97^***^	ns	–0.93^***^	0.90^***^	ns	ns	–0.98^***^
	Cd	–0.97^***^	ns	–0.97^***^	ns	ns	ns	ns	–0.98^***^	ns	0.99^***^	–0.98^***^	ns	ns	0.98^***^
	Ni	0.98^***^	ns	0.99^***^	ns	ns	ns	ns	0.97^***^	ns	–0.96^***^	0.93^***^	ns	ns	–0.96^***^
	Cr	ns	ns	–0.88^**^	ns	ns	ns	ns	–0.84^**^	–0.84	0.94^***^	–0.96^***^	ns	ns	0.77^*^
Fraction III	Zn	0.99^***^	ns	0.98^***^	ns	ns	ns	ns	0.98^***^	ns	–0.96^***^	0.94^***^	ns	ns	–0.97^***^
	Cu	0.92^***^	ns	0.97^***^	ns	ns	ns	ns	0.97^***^	ns	–0.98^***^	0.97^***^	ns	ns	0.94^***^
	Pb	–0.98^***^	ns	–0.96^***^	ns	ns	ns	ns	–0.98^***^	ns	0.91^***^	–0.87^**^	ns	ns	0.98^***^
	Cd	0.84^**^	ns	0.95^***^	ns	ns	ns	ns	0.87^**^	ns	–0.98^***^	0.99^***^	ns	ns	–0.86^**^
	Ni	0.90^**^	ns	0.98^***^	ns	ns	ns	ns	0.96^***^	ns	–0.97^***^	0.99^***^	ns	ns	0.92^***^
	Cr	0.74^*^	ns	0.88^**^	ns	ns	ns	ns	0.84^**^	0.84^**^	–0.94^***^	0.96^***^	ns	ns	ns
Fraction IV	Zn	0.98^***^	ns	0.97^***^	ns	ns	ns	ns	0.98^***^	ns	–0.92^***^	0.88^**^	ns	ns	–0.98^***^
	Cu	0.97^***^	ns	0.88^**^	ns	ns	ns	ns	0.91^***^	ns	ns	0.75^*^	ns	ns	–0.95^***^
	Pb	–0.83^**^	ns	–0.94^***^	ns	ns	ns	ns	–0.91^***^	ns	0.98^***^	–0.99^***^	ns	ns	0.85^**^
	Cd	0.99^***^	0.85^*^	0.99^***^	ns	ns	ns	ns	0.95^***^	ns	–0.97^***^	0.94^***^	ns	ns	ns
	Ni	0.98^***^	ns	0.96^***^	ns	ns	ns	ns	0.98^***^	ns	–0.91^***^	0.87^**^	ns	ns	–0.97^***^
	Cr	0.74	ns	0.88^**^	ns	ns	ns	ns	0.84^**^	0.84^**^	–0.94^***^	0.96^**^	ns	ns	–0.77^**^
PMF ∑I–Iii fractions	Zn	–0.98^***^	–0.78^*^	–0.94^***^	ns	ns	ns	ns	–0.94^***^	ns	0.92^***^	–0.88^***^	ns	ns	0.98^***^
	Cu	–0.97^***^	ns	–0.98^***^	ns	ns	ns	ns	–0.98^***^	ns	0.89^**^	–0.78^**^	ns	ns	0.95^***^
	Pb	0.83^**^	ns	0.94^***^	ns	ns	ns	ns	0.91^***^	0.74^**^	–0.98^***^	0.99^***^	ns	ns	–0.85^**^
	Cd	–0.97^***^	–0.87^**^	–0.98^***^	ns	ns	ns	ns	–0.96^***^	ns	0.91^***^	–0.87^**^	ns	ns	0.97^***^
	Ni	–0.97^***^	ns	–0.98^***^	ns	ns	ns	ns	–0.97^***^	ns	0.97^***^	–0.94^***^	ns	ns	0.98^***^
	Cr	0.72^**^	ns	0.87^**^	ns	ns	ns	ns	0.83^**^	0.85^**^	0.94^***^	–0.96^***^	ns	ns	ns
Total content	Zn	0.98^***^	0.69^**^	0.97^***^	ns	ns	ns	ns	0.98^***^	ns	–0.93^***^	0.89^**^	ns	ns	–0.98^***^
	Cu	ns	ns	–0.98^***^	ns	ns	ns	ns	–0.98^***^	ns	0.98^***^	–0.96^***^	ns	ns	0.98^***^
	Pb	0.74^**^	0.98^***^	0.56^*^	0.77^**^	0.56^*^	0.86^***^	0.92^***^	0.62^*^	ns	ns	ns	0.83^*^	0.94^***^	0.71^*^
	Cd	ns	ns	–0.64^**^	0.83^**^	0.96^***^	0.74^**^	0.64^*^	–0.58^*^	–0.98^***^	0.74^*^	ns	0.77^*^	ns	0.57^*^
	Ni	–0.97^***^	–0.66^*^	–0.98^***^	ns	ns	ns	ns	–0.98^***^	ns	0.95^***^	–0.91^***^	ns	ns	0.98^***^
	Cr	0.80^**^	0.98^***^	0.63^*^	0.71^**^	0.48	0.81^**^	0.88^**^	0.69^*^	ns	ns	ns	0.78^*^	0.91^***^	0.77^*^
PEC quotient		ns	0.90^***^	ns	0.98^***^	0.90^***^	0.98^***^	0.98^***^	ns	–0.85^**^	–	–	–	–	–

*IGe* inhibition of germination, *IR* inhibition of roots growth, *M* mortality, *IGr* inhibition of growth, *IL* inhibition of luminescence, *Ls* *L. sativum*, *Sa* *S. alba*, *Ss* *S. saccharatum*, *Hi* *H. incongurence*, *Vf* *V. fischeri*, *O* organic matter

Significant at * p ≤ 0.05; ** p ≤ 0.01, *** p ≤ 0.001, ns not significant

An analysis of correlation between the metal concentration in the sediments and the results of toxicity to the test organisms was also carried out (Table 5). Positive values of the correlation coefficients indicate a relation between metal concentration in the sediments and toxicity to organisms, whereas negative values might suggest that the concentration of a given metal in the sediments did not affect the sample toxicity. The highest number of positively significant correlations was found between the total concentration of Pb and Cr and germination and root growth inhibitions in the test plants as well as the mortality rate and growth inhibition in *H. incongruens*. Additionally, total Zn concentration correlated in a significantly positive way with germination inhibition in the test plants and with growth inhibition in *H. incongruens*. On the other hand, total Cd concentration correlated in a significantly positive way with root growth inhibition in the test plants and with the *H. incongruens* mortality. For Cu and Ni, most relations were negative. Generally, concentration of the metals in fraction I (MF) correlated in a significantly negative way with the response of the test organisms. It indicates that the concentration of the metals in fraction I did not affect their toxicity to the test organisms (Table 5). Only lead concentration in fraction I in some cases correlated positively with the response of the organisms (plant root growth inhibition, mortality of *H. incongruens*). However, this relation is not important due to a low concentration of this form of lead (below 1 % of the total concentration). Fraction II of Zn, Pb and Ni correlated positively with the germination inhibitions of *S. alba* and *S. saccharatum,* and with growth inhibition of *H. incongruens* (Table 5). On the other hand, fraction II of Cd, Cu and Cr was negatively correlated with the responses of some organisms. Results in Table 5 show that fractions III and IV of Zn, Cu, Cd, and Cr were positively correlated with germination inhibitions of *S. alba*, S*. saccharatum,L. sativum* (only Cd), growth inhibition of *H. incongruence,* and luminescence inhibition of *V. fischeri* (only Cr), while these fractions of Pb were negatively correlated with the response of the organisms. The potential metal fractions PMF ∑I–III showed a generally significant negative correlation with the toxicity of the samples (Table 5). Only the PMF of Pb and Cr in the studied sediments showed a significant positive correlation with the germination inhibition in *S. saccharatum and S. alba*, growth inhibition in *H. incong*ruens, and luminescence inhibition in *V. fischeri*.

## Discussion

Numerous studies have shown that content of clay fraction and of organic matter is the chief measure of the capacity of bottom sediments to accumulate contaminants (Farkas et al. 2007; Czerniawska-Kusza and Kusza 2011). Other important factors affecting metal mobility in sediment are: adsorption/desorption processes, salinity, presence of sulfur and carbonates, pH (Du Laing et al. 2009). Bottom sediments from the Rybnik reservoir had a high concentration of organic matter, which might have influenced the solubility and mobility of the heavy metals in the organisms. Organic matter has a high capacity to complex and adsorb cations due to the presence of numerous negatively charged groups. The studied sediments are rich in organic matter, therefore the formation of potential mobile metal-dissolved organic carbon complexes under oxidizing conditions prevents metals from co-precipitation with or adsorbing to Fe (hydr)oxides (Rinklebe and Shaheen 2014). In addition, from the studied fractions, the highest amount of Cu, Ni, Cr, Pb and Cd was associated with fraction III (organic), which indicates that this form of metals was dominant in sediments of the Rybnik reservoir. Positive significant correlations between the concentration of organic matter and the total metal concentration (r = 0.98 for Cu and Ni; 0.76 for Cr, 0.71 for Pb; and 0.57 for Cd, (p < 0.05)) confirm the considerable share of organic matter in the binding of heavy metals. Sediment organic matter also significantly affected the distribution of the metals among different geochemical fractions. Sand and silt content also significantly affected mobility and potential mobility of the metals. The positive relations of heavy metals with the organic matter concentration of the sediment might be attributed to anthropogenic impacts (Farkas et al. 2007; Shaheen and Rinklebe 2014). Of the studied metals, Zn has the highest mobility, since it presents the highest concentration in fraction I. The distribution of zinc is not unusual, high percentages of total zinc connected with more labile fractions in other studies of contaminated sediments have been found (Morillo et al. 2004; Hunglei et al. 2008). Most studies showed that soil or sediment pH is the key factor in determining Zn mobility (Hunglei et al. 2008; Shaheen and Rinklebe 2014). Results in Table 5 show that the distribution of Zn among different fractions did not significantly correlate with pH.

Both the analysis of total concentrations of metals and their fractionation showed that bottom sediments of the Rybnik reservoir are contaminated with heavy metals, which is generally an effect of intensive human impact of the area where reservoirs are located. Treated industrial sewage, emitted by the Rybnik power plant, municipal sewage, rain wastewaters, sewage from the water treatment plant, and cooling tower blowdowns are the main kind of water contamination in the reservoir and the Ruda River (main river which feeds the reservoir). The Ruda River itself is also a receiver of municipal sewage coming from municipal treatment plants and other industrial plants. Dry precipitation is another source of contamination of this water region; the Rybnik reservoir is located in an area where emissions of particulate and gas contaminants constitute approx. 19 % of total emissions in Poland. The second river, Nacyna, which flows into the reservoir, discharges sewage from Coal Company. Waters of the Nacyna River are transmitted by a pipeline bypassing the Rybnik reservoir, but insufficient capacity of the pumping station causes periodic overflowing of the Nacyna River directly to the reservoir. Studies by Loska and Wiechuła (2003) show that increases in Cd, Pb, Ni, and Zn concentrations in sediments were connected with an inflow of contaminated water of the Ruda River and long-range transport. Contamination of the sediment with Cu resulted mainly from atmospheric precipitation.

Bottom sediment quality assessment is often based on chemical and physical parameters without taking into account ecotoxicological investigations. Concentration of heavy metals alone does not provide sufficient information on the mobility and potential toxicity of contaminants or their potential harmful effects on the environment because different chemicals can inactivate and promote synergistic effects. In the studies, a potential harmful effect of metals on the environment was determined as a mean PEC quotient of six heavy metals. The mean PEC quotient provides a basis for assessing the potential effects of sediment-associated contaminants when they occur in a complex mixture (Perrodin et al. 2006). For each sediment sample, the mean PEC quotient was an average of the ratio of each metal concentration to its corresponding PEC. In this evaluation, sediment samples were predicted to be non-toxic if the mean PEC quotients were <0.5; otherwise, if >0.5, sediment samples were toxic (Hongyi et al. 2009). The mean PEC quotients of heavy metals have the following values: 1.2 (sediment sampling 1), 1.6 (sediment sampling 3) and 2.1 (sediment sampling 2). It means that all the sediment samples were potentially toxic. Potential toxicity of the bottom sediments was confirmed by toxicity results obtained from the performed battery of assays. The bottom sediment samples were classified as toxic and very toxic (class III, IV and V). Moreover, the value of PEC quotients correlated positively with plant root growth inhibition, germination inhibition of *L. sativum,* and mortality of *H. incongruens,* and negatively with luminescence inhibition of *V. fischeri* (Table 5). It is worth highlighting that the purpose of indices at PEC values is to distinguish concentrations that, when exceeded, are expected to lead to negative effects on organisms, and that methods of assessment of sediment quality using PEC indices and PEC quotients are based on the total concentrations of metals, not on their other forms. It is also commonly assumed that metals occurring in fraction I (MF, exchangeable) are mobile and available to living organisms and thereby can be toxic to them (Du Laing et al. 2009). The PMF (fraction II, III or PMF ∑1–3) of heavy metals is also considered as the potential hazardous fraction to organisms because this fraction is bound much less strongly with sediment solid phases than the residual fraction. The PMF constitutes the contaminated fraction that has the potential to enter into the mobile aqueous phase in response to changing environmental conditions (Knox et al. 2006; Rinklebe and Shaheen 2014). The studies did not confirm this dependence, because most correlation dependencies between the MF, PMF ∑1–3 and the response of the organisms were negative (without Pb and Cr—PMF) or insignificant, which means that the sediment toxicity to the test organisms did not depend on the metals concentration in these fractions. On the other hand, the MF and PMF ∑1–3 showed a significant positive correlation with organic matter. This means that the studied sediments reveal a risk concerning metal pollution because metals associated with organic matter are likely to be released if the organic matter is decomposed and oxides e.g. under aerobic conditions (Shaheen and Rinklebe 2014).

In this study, the samples of bottom sediments, sediment elutriates and pore water toxicity were assessed using a battery of bioassays. In other authors’ studies, only one or two organism species were used to evaluate the toxicity of bottom sediments (Czerniawska-Kusza et al. 2006; Baran and Tarnawski 2013; Goncalves et al. 2013). Latif and Licek (2004) described the need to apply a battery of bioassays for an integral and ecologically meaningful assessment of the hazard associated with the presence of various chemical substances in water and bottom sediments, which could endanger organisms living in these environments. The analysis of results obtained from the conducted bioassays indicates that it seems necessary, however, to apply a battery of bioassays that uses organisms from different tropic levels and with different sensitivity to substances present in sediments. It is important that each species and test procedure have their own sensitive pattern to toxicants, no single species is sensitive to all chemicals (Matejczyk et al. 2011). The use of a variety of organisms representing different trophic levels, habitat and sensitivity to toxicants, allows one to assess the potential of toxic contaminants, considering several exposure routes (whole sediment, pore water) and different endpoint effects based on sensitivity (Narracci et al. 2009). In the studies, the test organisms were exposed to substances dissolved in water (Microtox, Phytotestkit) as well as to substances absorbed on the surface of solid particles (Phytotoxkit, Ostracodtoxkit F). It is important since a large part of toxic substances, including heavy metals, undergo sorption on non-organic and organic particles, and is available only during direct contact (Leitgib et al. 2007; Plaza et al. 2010). The studies showed that sediments from the Rybnik reservoir are toxic, but the used organisms showed different sensitivity to heavy metals occurring in the bottom sediments (Fig. 3; Table 5). The correlation analysis revealed certain relations; firstly, the lack of significant correlation between the fractions of heavy metals (Zn, Cu, Cd, Ni, Cr) in the sediments and root growth inhibition in tested plants, and mortality of *H. incongruens*. Secondly, germination inhibition of *L. sativum* and luminescence inhibition of *V. fischeri* also did not show a lot of significant relationships with fractions of metals. Thirdly, the strongest significant positive correlations were found between fractions III (forms bound to organic matter) and IV (residual fraction) of the metals and toxicity to organisms. A positive correlation was also recorded between total concentration of Zn, Pb, Cr and germination inhibition, growth inhibition of *H. incongruens*; Pb, Cd, Cr and plant root growth inhibition, mortality of *H. incongruens.* Lack of significant correlation between heavy metal concentration in sediments and response of organisms suggests that there are other factors, not measured in this study, that contribute to the toxicity of the analyzed sediments, such as PAHs and PCB concentration in sediment. In other authors’ studies it was found that *H. incongruens* is the most appropriate test to detect toxicity in hydrocarbon-contaminated soil samples (Płaza et al. 2010). Additionally, interactions between contaminants may result in antagonistic or synergistic effects that are difficult to predict (Czerniawska-Kusza et al. 2006; Simeonov et al. 2007). Moreover, the studies of Simeonov et al. (2007) showed that the assessment of some simple relations between chemical parameters and ecotoxicity of sediments did not give any hint of serious correlation between them. Sediment assessment needs to be done in two separate analytical procedures—chemical and ecotoxicological, which use bioassays to receive information about acute and chronic toxicity (Simeonov et al. 2007). The above-mentioned factors can also explain the relatively low toxicity of bottom sediments collected in point 2, despite the fact that sediments from that point were characterized by the highest total concentration of metals as well as their highest mobility among the studied measuring points. The studies showed a higher toxicity in the bottom sediments than in pore water. The high toxicity of the sediment samples was not always accompanied by an associated toxicity of the pore water. The studied samples of pore water revealed a negative value of toxicity for *V. fischeri*. It means that the luminescence intensity of *V. fischeri* increased after exposure to pore water as a result of their lower toxicity compared to the control medium. The stimulation of light emission has been observed by other authors, and classified under the term hormesis (Christofi et al. 2002). Samples showing hormesis are currently non-toxic. Compared to *V. fischeri*, greater pore water toxicity, especially for plants (germination), was caused by the concentration of ammonia in the pore water. Ammonia concentration in the pore water was not analyzed in the study. However, in other papers it has been found that pore water ammonia concentrations can be inhibitory to test organisms (Phillips et al. 2005; Rosen et al. 2008; Łukawska-Matuszewska et al. 2009). Other studies have found that the Microtox^®^ test is an inadequate indicator of ammonia toxicity since it is not very sensitive to ammonia (Rosen et al. 2008). The assessments of the pore water toxicity (containing water-soluble and labile compounds) as well as the toxicity of the solid phase of the sediments (which is usually associated with water-insoluble contaminants) allow one to assess which forms of contaminants are responsible for sediment toxicity (Goncalves et al. 2013). The studies revealed that the toxicity of the sediments is caused mainly by heavy metal forms associated with the solid phase of the sediments.

## Conclusion

The analysis of total concentrations of metals and their fractionation showed that sediments of the Rybnik reservoir are contaminated with heavy metals, which is an effect of intensive human impact of the area where the reservoir is located. Organic matter is likely to be an important factor which controls metal distribution and mobility in the studied sediments. The highest amount of Cu, Ni, Cr, Pb and Cd was associated with fraction III (organic), which indicates that this form of metals was dominant in sediments of the Rybnik reservoir. Sand and silt also significantly affected the distribution of metals among different geochemical fractions. Sediments from the Rybnik reservoir were toxic, but the used organisms showed different sensitivity. Moreover, the studies showed a higher toxicity in solid phases and whole sediment than in the pore water. The highest number of toxic responses was recorded for *L. sativum* (whole sediment, solid phases) as well as for *V. fischeri* and *S. alba* (solid phases), and for *S. saccharatum* and *L. sativum* (pore water). Among all the test organisms, the lowest sensitivity was observed in *H. incongruens* (solid phases*)* and *V. fischeri* (pore water, whole sediment). The toxicity of the sediments is caused mainly by metal forms associated with the solid phase of the sediments. In the studies, the most significant positive correlations were found between fractions III (forms bound to organic matter) and IV (residual fraction) of the metals and toxicity to organisms. Additionally, the total metal concentrations correlated in a significantly positive way with the response of the test organisms. The PEC quotient of the six metals, which assess the potential effects of sediment-associated contaminants when they occur in a complex mixture, correlated positively with plant root growth inhibition, germination inhibition of *L. sativum* and mortality of *H. incongruens*.

In conclusion, in order to identify the degree of contamination of sediments, it is important to conduct, next to chemical analyses, an ecotoxicological classification of sediment quality using a battery of bioassays as biosensors for changes in the water ecosystem. A multitrophic battery of different test species allows toxicity levels to be correctly evaluated, reducing the uncertainty in sediment quality assessments.
